# Genome-wide identification and expression analysis of *TPS* gene family contributing to cold tolerance in *Actinidia arguta*

**DOI:** 10.3389/fpls.2026.1894816

**Published:** 2026-07-30

**Authors:** Yunqing Liu, Guangli Shi, Dan Sun, Zhenxing Wang, Chengcheng Zhao, Jun Ai

**Affiliations:** 1 College of Life Science, Jilin Agricultural University, Changchun, China; 2 College of Horticulture, Jilin Agricultural University, Changchun, China

**Keywords:** *Actinidia arguta*, cold tolerance, gene family, subcellular localization, trehalose-6-phosphate synthase (TPS)

## Abstract

Trehalose-6-phosphate synthase (TPS) plays an important role in the response of plants to abiotic stress. To explore the characteristics of the *TPS* gene family in *Actinidia arguta* and its expression levels in cold tolerance, this study identified *TPS* gene family members, analyzed their physicochemical properties, chromosomal localization, phylogenetic relationships, conserved motifs, gene structure, cis-regulatory elements, and synteny relationships, and examined their expression levels on overwintering. The results showed that a total of 16 *TPS* genes were identified in the *A. arguta*, randomly anchored on 12 chromosomes, with amino acid lengths ranging from 414 to 936 aa, all of which are hydrophilic proteins. Based on phylogenetic analysis, all *TPS* genes were divided into two classes, and the promoter of the *AaTPS* is densely distributed with cis-elements related to hormone response, growth and development, and stress response. Combining transcriptome data with qRT-PCR analysis, *AaTPS8* was identified, which is closely related to the overwintering of *A. arguta*. Further subcellular localization results indicate that the AaTPS8 protein is primarily localized in the cytoplasm, suggesting its potential involvement in trehalose metabolism and stress response regulation in the cytoplasm. The results of this study provide new insights into understanding the potential functions of *TPS* genes under stress conditions and offer a theoretical genetic basis and important reference for breeding *A. arguta* cultivars that can adapt to cold tolerance.

## 1 Introduction

Trehalose-6-phosphate (T6P) is an important stage in the trehalose biosynthesis pathway and has recently been identified as a key sugar signaling molecule in plants. T6P functions as a metabolic sensor that closely synchronizes plant growth and developmental programs with sucrose status (Lunn et al., 2014; Paul and Pellny, 2003; Wahl et al., 2013; Zhang et al., 2009). By regulating the carbon-nitrogen balance in plants, T6P plays a crucial function in metabolic homeostasis and physiological adaptation in addition to its roles in carbon allocation and developmental regulation (Fichtner and Lunn, 2021; Figueroa and Lunn, 2016; Nunes et al., 2013). As the rate-limiting enzyme in this pathway, trehalose-6-phosphate synthase (TPS) catalyzes the irreversible conversion of uridine diphosphate glucose (UDP-glucose) and glucose-6-phosphate into T6P, the initial committed step in trehalose biosynthesis. TPS thus holds a crucial and indispensable regulatory role in regulating plant development, growth, and stress tolerance (Lunn et al., 2014; Schluepmann et al., 2003). Notably, higher plants only have trace amounts of trehalose, unlike microbes that increase stress tolerance by massively accumulating trehalose. Instead, the biological functions of the trehalose pathway in plants are primarily mediated by T6P itself and its regulatory interplay with the SNF1-related protein kinase 1 (SnRK1) signaling pathway, rather than by the accumulation of trehalose as an end product (Zhang et al., 2009; Figueroa et al., 2016; Paul et al., 2018; Ponnu et al., 2011).

In higher plants, *TPS* typically exists as a multigene family and has undergone extensive expansion and functional diversification during plant evolution. In the model plant *Arabidopsis thaliana*, for example, a total of 11 *TPS* genes have been identified in the genome. Based on differences in protein structural integrity and enzymatic activity, these genes can be classified into two distinct classes. Class I TPS proteins possess complete catalytic activity and function as the primary enzymes responsible for T6P biosynthesis, whereas Class II TPS proteins retain conserved structural domains but generally lack catalytic activity and are therefore thought to play roles in regulatory processes or signal transduction rather than direct enzymatic function (Leyman et al., 2001; Dijken et al., 2004; Vandesteene et al., 2010). According to phylogenetic investigations, the *TPS* gene family first appeared early in the history of terrestrial plants. As monocotyledonous and dicotyledonous lineages split apart, it underwent several rounds of gene duplication and functional divergence. Significant interspecific heterogeneity in *TPS* gene number, structural characteristics, and expression patterns across several plant species has been caused by these evolutionary events (Lunn et al., 2014; Acosta-Pérez et al., 2020; Gao et al., 2021; Zang et al., 2011). All of the genome-wide research points to the *TPS* gene family’s diversification as a key molecular foundation for precisely regulating carbon signaling and enabling plants to respond to complex and changing environmental situations.

A growing amount of data suggests that the T6P-TPS-SnRK1 signaling module is essential for controlling how plants react to abiotic stressors, especially cold stress. Low-temperature stress causes significant disruptions in plant carbon allocation and energy homeostasis. T6P, a major sugar signaling molecule, regulates the activity of SNF1-related protein kinase 1 (SnRK1), altering carbon metabolic pathways and transcriptional regulatory networks involved in stress responses (Figueroa et al., 2016; Krasensky and Jonak, 2012). *TPS* genes in numerous plant species show considerable activation or repression under low-temperature circumstances, and these expression variations are directly related to cold tolerance metabolic adaptations. Genetic modification of *TPS* gene expression has been shown in numerous model plants and crops to improve tolerance to abiotic challenges, emphasizing the importance of T6P-mediated sugar signaling pathways in stress adaption (Paul et al., 2018, 2020). However, current research on the role of *TPS* genes in cold stress responses has been focused on annual herbaceous plants, with few systematic investigations into perennial woody species.

*Actinidia arguta* is one of the most cold-tolerant species in the *Actinidia* genus, able to tolerate exceptionally low temperatures of -30°C or lower, making it a suitable system for studying cold tolerance processes in fruit trees. In recent years, as genomic resources for *Actinidia* species have improved, there has been a greater emphasis on understanding the molecular regulatory mechanisms controlling growth, development, and stress responses. Despite the fact that the *TPS* gene family has been described in *A. thaliana* (Vandesteene et al., 2010), watermelon (Yuan et al., 2021), cucumber (Dan et al., 2021), apple (Du et al., 2017), and peach (Fan et al., 2024), a thorough examination of this gene family in *A. arguta* has not yet. Based on the reference genome of *A. arguta*, the *TPS* gene family was identified genome-wide in this investigation. The physicochemical properties, gene structures, conserved motif compositions, and phylogenetic relationships of the encoded TPS proteins were systematically analyzed using bioinformatic approaches. Furthermore, transcriptome data were integrated to elucidate the expression patterns of *AaTPS*s family members during the overwintering process in *A. arguta*, and their transcriptional responses were further validated by qRT-PCR analysis. This study is expected to deepen our understanding of sugar signal-mediated cold tolerance mechanisms in woody fruit trees and to provide valuable candidate genes and a theoretical foundation for molecular breeding of cold-tolerant *A. arguta* cultivars.

## 2 Materials and methods

### 2.1 Identification and physicochemical property analysis of *AaTPS*s

The genome assembly and annotation resources of *A. arguta* were obtained from the Plant GARDEN database (https://plantgarden.jp/ja/list/t1837144/genome/t1837144.G001). The genome assembly AAR_r2.2, together with the corresponding genome sequence, protein FASTA, CDS and GFF3 annotation files, was used for *TPS* gene identification, sequence retrieval, chromosomal localization and gene-structure analysis. *AaTPS* family members were identified by combining BLASTP searches and hidden Markov model (HMM). The protein sequences of *A. thaliana* TPS proteins (AtTPS1: At1g78580; AtTPS2: At1g16980; AtTPS3: At1g17000; AtTPS4: At4g27550; AtTPS5: At4g17770; AtTPS6: At1g68020; AtTPS7: At1g06410; AtTPS8: At1g70290; AtTPS9: At1g23870; AtTPS10: At1g60140; AtTPS11: At2g18700) were used as queries for BLASTP searches against the *A. arguta* protein database using the BLAST function implemented in TBtools v2.471. The E-value threshold was set to 1 × 10^-5^, and all other parameters used default values. Candidate TPS proteins were further identified using HMMER v3.0 with the Pfam HMM profiles PF00982 (Glyco_transf_20) and PF02358 (Trehalose_PPase) using an E-value cutoff of 1 × 10^-5^. Conserved domains were subsequently confirmed using SMART and Pfam online website. When multiple transcript isoforms were present for the same gene locus, only the longest protein isoform was retained for subsequent analyses.

After removing redundant sequences, the final members of the *AaTPS* gene family were identified. The physicochemical properties of TPS proteins, including amino acid length (AA), molecular weight (MW), theoretical isoelectric point (pI), instability index (II), aliphatic index (AI), and grand average of hydropathicity (GRAVY), were predicted using the ExPASy ProtParam online tool (https://web.expasy.org/protparam). The secondary and tertiary structures of TPS proteins were predicted using the Prabi server and SWISS-MODEL (https://swissmodel.expasy.org/), respectively.

### 2.2 Phylogenetic analysis of the *AaTPS* gene family

TPS protein sequences from *A. arguta* and *A. thaliana*, rice (Zang et al., 2011), apple (Du et al., 2017), and peach (Fan et al., 2024) were selected for phylogenetic analysis. Multiple sequence alignment of TPS proteins was first performed using Clustal W implemented in MEGA 12 software with default parameters. A phylogenetic tree was subsequently constructed using the Maximum Likelihood (ML) method with 1,000 bootstrap replicates to evaluate branch support. The Jones-Taylor-Thornton (JTT) model was selected as the amino acid substitution model, and positions containing gaps or missing data were treated using the partial deletion option. The classification was performed with reference to the *AtTPS* gene family. The resulting phylogenetic tree was visualized and annotated using the online tool iTOL.

### 2.3 Domain architecture, gene structure, and conserved motif analysis of the *AaTPS* gene family

The conserved domains of TPS proteins were annotated using the Pfam and SMART databases. Exon-intron structural information of *AaTPS*s was extracted from the *A. arguta* genome annotation (GFF3) file, and the domain architecture and gene structures were visualized using TBtools software. Conserved motifs among TPS proteins were identified using the MEME online tool (https://meme-suite.org/meme/), with the maximum number of motifs set to 10 and other parameters set to default values. The identified motifs were subsequently visualized using the iTOL online platform (https://itol.embl.de/).

### 2.4 Chromosomal localization, synteny, and Ka/Ks analysis of the *AaTPS* gene family

Chromosomal location information of *AaTPS*s was retrieved from the genome annotation file and visualized using the online tool MG2C (http://mg2c.iask.in/mg2c_v2.1/). Gene duplication and synteny analyses were performed using the MCScanX implemented in TBtools v2.471. Protein sequences were subjected to an all-versus-all BLASTP search with an E-value cutoff of 1 × 10^-5^, and the resulting BLASTP output together with the corresponding gene position (GFF) file were used as input for MCScanX. Default MCScanX parameters were applied, with the minimum number of genes required to define a collinear block set to 5. In addition, the *A. thaliana* genome was used as a reference for interspecies synteny analysis with *A. arguta* to investigate the evolutionary conservation of the *TPS* gene family. All syntenic relationships were visualized using TBtools. Based on the identified homologous gene pairs, coding sequence (CDS) alignments were extracted, and the nonsynonymous substitution rate (Ka), synonymous substitution rate (Ks), and their ratio (Ka/Ks) were calculated using the built-in programs in TBtools to evaluate the selective pressure acting on *AaTPS* genes during evolution. CDS alignment, Ka/Ks calculation and screening of duplication events were all performed using the KaKs_Calculator module in TBtools with default parameters.

### 2.5 Cis-acting element analysis of *AaTPS*s promoters

Based on the *A. arguta* genome annotation file, 2,000 bp upstream genomic sequences from the transcription start site of each *AaTPS*s were extracted as promoter regions. These promoter sequences were submitted to the PlantCARE database for prediction and annotation of cis-acting regulatory elements. The distribution of cis-elements was visualized using the Simple BioSequence Viewer module in TBtools.

### 2.6 Plant materials, transcriptome, RNA extraction, and qRT-PCR analysis

One-year-old branches of two *A. arguta* cultivars with contrasting cold tolerance were used as experimental materials: the cold-tolerant cultivar ‘Jialv’ (‘J’) and the relatively cold-sensitive cultivar ‘Longcheng No. 2’ (‘L’). The plant materials were grown in the *A. arguta* germplasm repository of Jilin Agricultural University, a standardized and well-managed germplasm orchard with a single-row parallel planting system. Mature plants of *A. arguta* were selected, and samples were consistently collected from the middle portion of one-year-old branches with a diameter of approximately 0.6 cm. For each cultivar, five healthy plants with uniform growth and no visible pests or diseases were selected as biological replicates. Samples were collected on September 16 (T1), October 6 (T2), November 6 (T3), and December 6 (T4) in 2022. The field temperature data recorded on each sampling date are listed in **Table 1**. All samples were immediately frozen in liquid nitrogen and stored at -80°C for subsequent transcriptome sequencing and quantitative real-time PCR (qRT-PCR) analyses.

**Table 1:** Field daily temperatures for each sampling date in 2022.

Date	Lowest temperatures (°C)	Highest temperatures (°C)
September 16 (T1)	14.18	17.42
October 6 (T2)	-1.30	11.22
November 6 (T3)	-2.60	10.98
December 6 (T4)	-13.18	-4.57

For transcriptome analysis, sequencing was performed using the Illumina NovaSeqTM 6000 platform, and the reference genome index was constructed using HISAT2 (v2.2.1). The mapped reads of each sample were assembled using StringTie (v2.1.6) with default parameters. After the final transcriptome was generated, StringTie and ballgown (v0.9.8) were used to estimate the expression levels of all transcripts and perform expression abundance for mRNAs by calculating FPKM (fragment per kilobase of transcript per million mapped reads) value. The expression matrix based on standardized FPKM values was used for further expression pattern analysis, which was visualized using the TBtools software. All samples of the two cultivars at each stage were collected with three biological replicates.

Total RNA was extracted using Plant RNA Extraction Kit (GeneStar, P134-01). First-strand cDNA was synthesized using the TransScript All-in-One First-Strand cDNA Synthesis SuperMix (GeneStar, A230-10). Gene-specific primers were designed using Primer Premier 5.0. Actin (ID: Aar2.2ch23g30510.1) was used as the internal reference gene. qRT-PCR was performed using a SYBR Green-based assay. The total reaction volume was 20 μL, consisting of 10 μL of 2× Universal SYBR qPCR Mix (Biosharp, BL697A), 0.5 μL of each forward and reverse primer (10 μM), 1 μL of cDNA template, 0.4 μL of ROX Reference Dye and 7.6 μL of RNase-Free ddH_2_O. The PCR program consisted of an initial denaturation at 95°C for 2 min, followed by 40 cycles of 95°C for 15 s and 60°C for 30 s. A melt curve analysis was performed after amplification to verify the specificity of the PCR products. Each sample was analyzed in three biological replicates. Relative gene expression levels were calculated using the 2^^-ΔΔCt^ method. Data are presented as the mean ± standard error.

### 2.7 Subcellular localization of *AaTPS8*

The coding sequence (CDS) of AaTPS8 without the native stop codon was amplified from A. arguta cDNA using gene-specific primers (Forward primer: ATGACGGTTCCGGGCATAATTTCG; Reverse primer: AGATCCTCCTCCAGATCCTCCTCCCCACTGGACTTCATGTTTTATCG), followed by seamless cloning. The amplified fragment was cloned into the plant expression vector pBWA(V)H2S to construct a C-terminal GFP fusion construct (35S::AaTPS8-GFP) driven by the CaMV 35S promoter. The recombinant plasmid was verified by DNA sequencing. The recombinant plasmid was introduced into Agrobacterium GV3101 by electroporation. Agrobacterium was cultured at 25 °C for 2 days. Single colonies were inoculated into 10 mL of YEB medium containing kanamycin. Cells were centrifuged, resuspended in infiltration buffer, and adjusted to an OD600 of 0.6. For subcellular localisation analysis, healthy *Nicotiana benthamiana* plants aged 4–6 weeks were selected for transient expression. The Agrobacterium suspension carrying 35S::AaTPS8-GFP was mixed with a suspension carrying the cytoplasmic marker construct 35S::HSBP-mKate at a 1:1 ratio and infiltrated into the abaxial surface of Nicotiana benthamiana leaves using a needleless syringe. The empty vector 35S::GFP was used as a negative control. After infiltration, the plants were cultured under low-light conditions for 48 hours. T The infiltrated tobacco leaves were observed using a laser scanning confocal microscope (Nikon C2). Three biological replicates were performed.

## 3 Results

### 3.1 Identification and physicochemical characterization of the *AaTPS* gene family

Based on BLAST and HMM approaches, a total of 16 *TPS* genes were identified in the *A. arguta* genome and were designated as *AaTPS1* to *AaTPS16* according to their chromosomal locations. Physicochemical property analysis revealed that the *AaTPS* family members encoded proteins ranging from 414 to 936 amino acids in length (**Table 2**). The predicted molecular weights varied from 46921.88 Da (AaTPS5) to 106062.46 Da (AaTPS1), while the theoretical isoelectric points (pI) ranged from 5.52 (AaTPS6) to 9.24 (AaTPS13). The instability index of AaTPS proteins ranged from 34.61 (AaTPS11) to 47.94 (AaTPS3). Most members (14 out of 16) were predicted to be unstable proteins (instability index > 40), whereas only AaTPS11 and AaTPS13 were classified as stable. The aliphatic index ranged from 82.35 (AaTPS10) to 94.04 (AaTPS12). All AaTPS proteins exhibited negative GRAVY values (-0.157 ~ -0.350), indicating that they are hydrophilic in nature.

**Table 2:** Physicochemical property analysis of *AaTPS* gene family.

Gene name	Gene ID	Chr	start site	End site	Strand direction	Amino acid	Molecular weight	Isoelectric point	Instability index	Aliphatic index	Grand average of hydropathicity
*AaTPS1*	Aar2.2ch06g13410.1	6	12534488	12554957	+	936	106062.46	7.72	41.47	90.19	-0.302
*AaTPS2*	Aar2.2ch06g13770.1	6	12802909	12806540	–	855	96469.35	5.65	47.31	85.72	-0.185
*AaTPS3*	Aar2.2ch12g20940.1	12	22205939	22209597	+	857	97167.28	5.85	47.94	84.28	-0.265
*AaTPS4*	Aar2.2ch13g02950.1	13	2587965	2591071	–	863	98103.52	6.39	41.63	89.54	-0.205
*AaTPS5*	Aar2.2ch16g15040.1	16	14298698	14300206	+	409	46921.88	8.05	40.33	87.19	-0.324
*AaTPS6*	Aar2.2ch17g01690.1	17	1507452	1511642	–	857	97242.29	5.52	47.68	84.28	-0.265
*AaTPS7*	Aar2.2ch17g06160.1	17	5963821	5979403	+	933	104999.36	6.54	42.48	85.89	-0.350
*AaTPS8*	Aar2.2ch18g14160.1	18	12367246	12369623	–	697	79542.08	6.02	46.66	88.22	-0.184
*AaTPS9*	Aar2.2ch18g23160.1	18	21198221	21201428	+	856	96217.97	5.92	43.86	84.80	-0.213
*AaTPS10*	Aar2.2ch22g17190.1	22	17617411	17619060	+	425	48468.63	8.86	44.01	82.35	-0.307
*AaTPS11*	Aar2.2ch23g32480.1	23	31568323	31571928	–	734	82770.91	5.70	34.61	90.49	-0.162
*AaTPS12*	Aar2.2ch25g06760.1	25	7767083	7769795	+	804	91623.35	5.95	44.88	94.04	-0.204
*AaTPS13*	Aar2.2ch25g16550.1	25	17895751	17897271	–	414	47107.40	9.24	39.34	88.55	-0.253
*AaTPS14*	Aar2.2ch26g08470.1	26	8116670	8118664	–	664	74872.83	6.24	43.30	92.30	-0.157
*AaTPS15*	Aar2.2ch27g08650.1	27	8507784	8511332	+	854	96680.04	5.54	42.28	90.94	-0.182
*AaTPS16*	Aar2.2ch29g04240.1	29	4316579	4319714	+	878	99852.56	6.09	47.62	89.01	-0.207

### 3.2 Phylogenetic analysis of the *AaTPS* gene family

**Figure 1:**
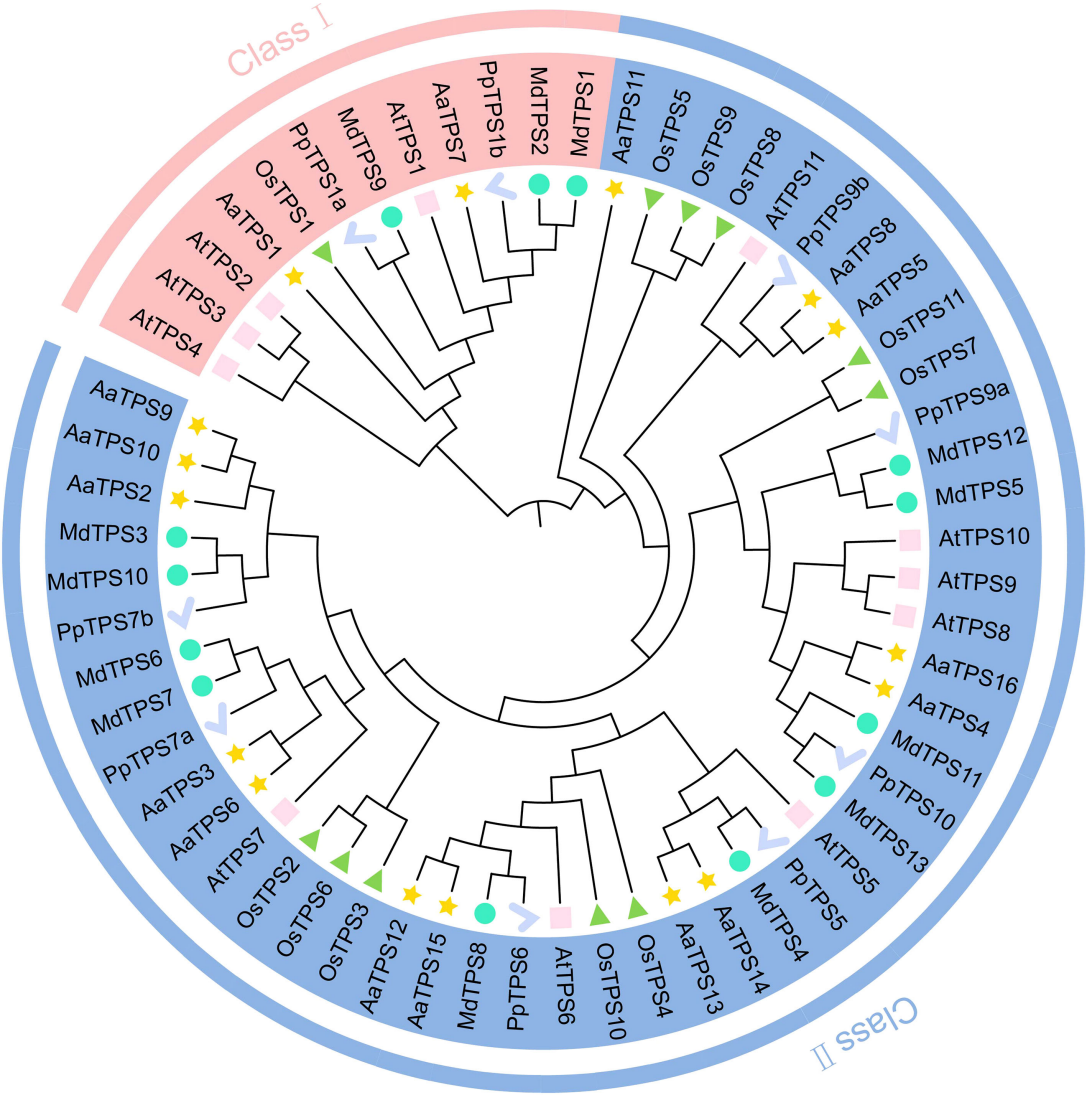
Phylogenetic tree of *A. arguta* and other species *TPS*s. Classes I and II are denoted by pink and blue, respectively. Yellow stars represent *AaTPS*s, pink squares represent *AtTPS*s, green triangles represent *OsTPS*s, green circles represent *MdTPS*s, and purple check marks represent *PpTPS*s.

To investigate the evolutionary relationships of the *AaTPS* gene family, a phylogenetic tree was constructed based on the full-length amino acid sequences of TPS proteins from *A. arguta*, *A. thaliana*, rice (*O. sativa*), apple (*M. domestica*), and peach (*P. persica*). A total of 60 TPS protein sequences were included in the analysis. Phylogenetic analysis showed that all TPS proteins were clearly classified into two major clades, designated as Class I and Class II (**Figure 1**). The Class I clade consisted of 12 individuals, including two from *A. arguta*, four from *A. thaliana*, one from rice, three from apple, and two from peach. The Class II clade was assigned to the remaining 48 TPS proteins. Class I *TPS* genes were comparatively rare and well conserved among various species from the standpoint of species distribution. Interestingly, just two *AaTPS*s (*AaTPS1* and *AaTPS7*) were classified as Class I, suggesting that the Class II clade was primarily responsible for the expansion of the *AaTPS* gene family.

### 3.3 Conserved domain architecture, gene structure, and motif analysis of AaTPS proteins

**Figure 2:**
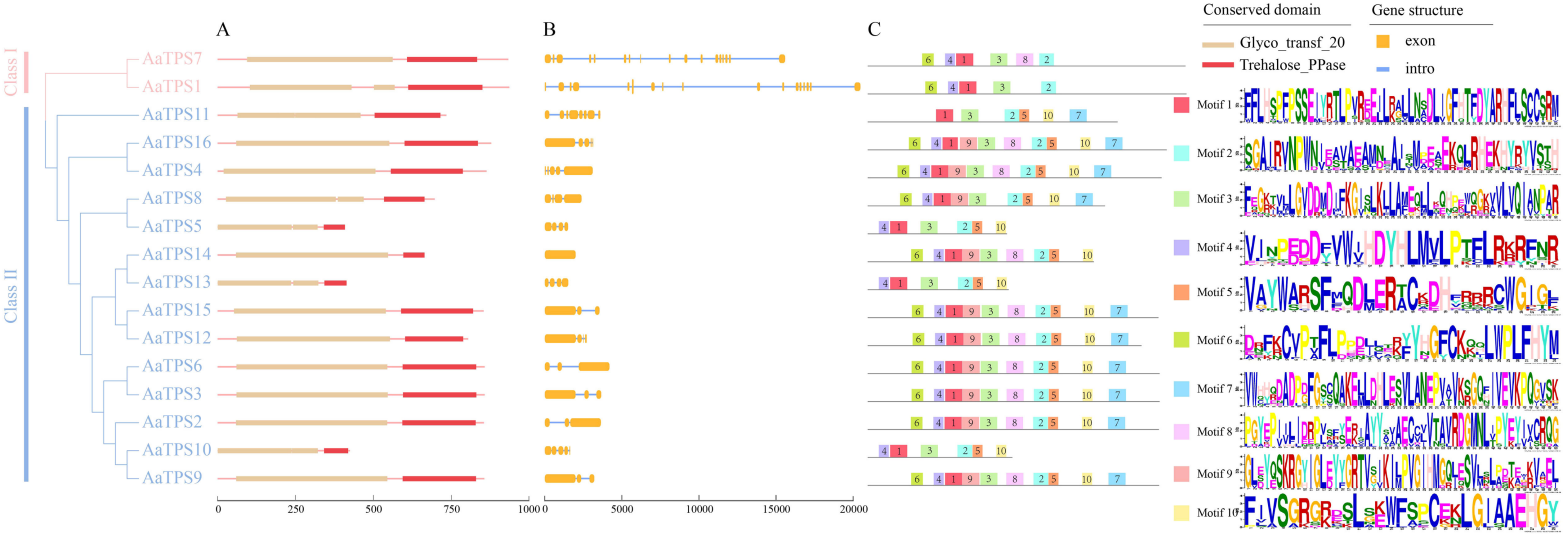
Conserved domain **(A)**, gene structure **(B)** and motif compositions **(C)** of *TPS* genes in *A. arguta*.

Conserved domain analysis was carried out on each protein to better describe the structural characteristics of AaTPS proteins (**Figure 2A**). The findings demonstrated that the distinctive TPS family domains PF00982 (Trehalose-6-phosphate synthase) and PF02358 (Trehalose-phosphatase) were present in all AaTPS proteins, suggesting a high level of structural conservation among AaTPS proteins. According to the usual structural characteristics of plant TPS proteins, AaTPS1 and AaTPS7 were moderately lengthy proteins (>900 aa) with a wide distribution of the PF00982 domain and the PF02358 domain at the C-terminus. AaTPS5, AaTPS10, and AaTPS13, which are shorter proteins (around 400 aa), on the other hand, showed fragmented or repeated distributions of the PF00982 domain in addition to a comparatively truncated PF02358 domain. These structural characteristics imply that these individuals might have experienced functional diversification or structural simplification throughout evolution.

**Figure 3:**
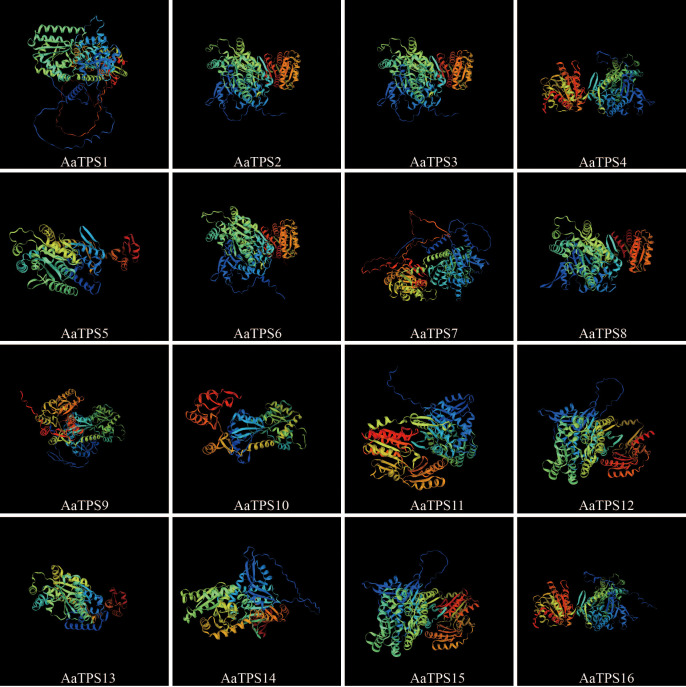
The protein tertiary structure of AaTPSs.

According to secondary structure prediction (**Table 3**), the majority of the 16 AaTPS proteins were made up of random coils and α-helices, whereas the percentage of extended strands was steady and relatively low. Overall, AaTPS proteins exhibited preserved secondary structural features, with α-helix content ranging from 36.00% to 50.61%, extended strands from 13.83% to 16.18%, and random coils from 33.57% to 49.79%. The tertiary structure of AaTPS protein is shown in **Figure 3**.

**Table 3:** Secondary structure of AaTPS proteins.

Gene name	Alpha helix	Extended strand	Random coil
AaTPS1	36.00%	14.21%	49.79%
AaTPS2	42.81%	14.62%	42.57%
AaTPS3	42.24%	14.47%	43.29%
AaTPS4	44.26%	14.60%	41.14%
AaTPS5	50.61%	15.16%	34.23%
AaTPS6	42.47%	14.70%	42.82%
AaTPS7	41.80%	13.83%	44.37%
AaTPS8	44.19%	15.78%	40.03%
AaTPS9	44.28%	14.84%	40.89%
AaTPS10	50.35%	15.29%	34.35%
AaTPS11	42.23%	15.40%	42.37%
AaTPS12	42.79%	13.93%	43.28%
AaTPS13	50.24%	16.18%	33.57%
AaTPS14	43.52%	14.61%	41.87%
AaTPS15	43.09%	15.34%	41.57%
AaTPS16	43.17%	14.35%	42.48%

The gene structural architecture of *AaTPS*s was viewed by analyzing exon-intron structures (**Figure 2B**). The results demonstrated that AaTPSs varied considerably in terms of gene length and exon count. Signs of complex gene architectures were found in *AaTPS1* and *AaTPS7*, which both had 17 exons and relatively long gene lengths of 20469 bp and 15582 bp. AaTPS14, however, contains a single exon and a relatively basic gene structure. While certain members (including *AaTPS1*, *AaTPS7*, and *AaTPS11*) showed a discernible increase in exon number, suggesting that exon gain or intron insertion events occurred during evolution, most *AaTPS*s had three to six exons and displayed relatively intact structural patterns. Exon-intron architectures of *AaTPS*s with closer phylogenetic ties frequently tended to be more similar, further demonstrating their evolutionary and functional relatedness.

Several highly conserved motifs were found across AaTPS proteins by motif analysis; the majority of members shared a core motif composition and showed essentially similar motif configurations within the same evolutionary group (**Figure 2C**). These conserved motifs are strongly linked to the catalytic activity of TPS proteins and were mostly found in the PF00982 and PF02358 domain regions. Interestingly, shorter AaTPS proteins like AaTPS5, AaTPS10, and AaTPS13 either have fewer motifs or no motifs at all, indicating that they could not have full catalytic activity but rather be engaged in signaling or regulatory processes. This observation aligns with their gene structural features and domain makeup.

### 3.4 Chromosomal localization, synteny, and Ka/Ks analysis of the *AaTPS* gene family

**Figure 4:**
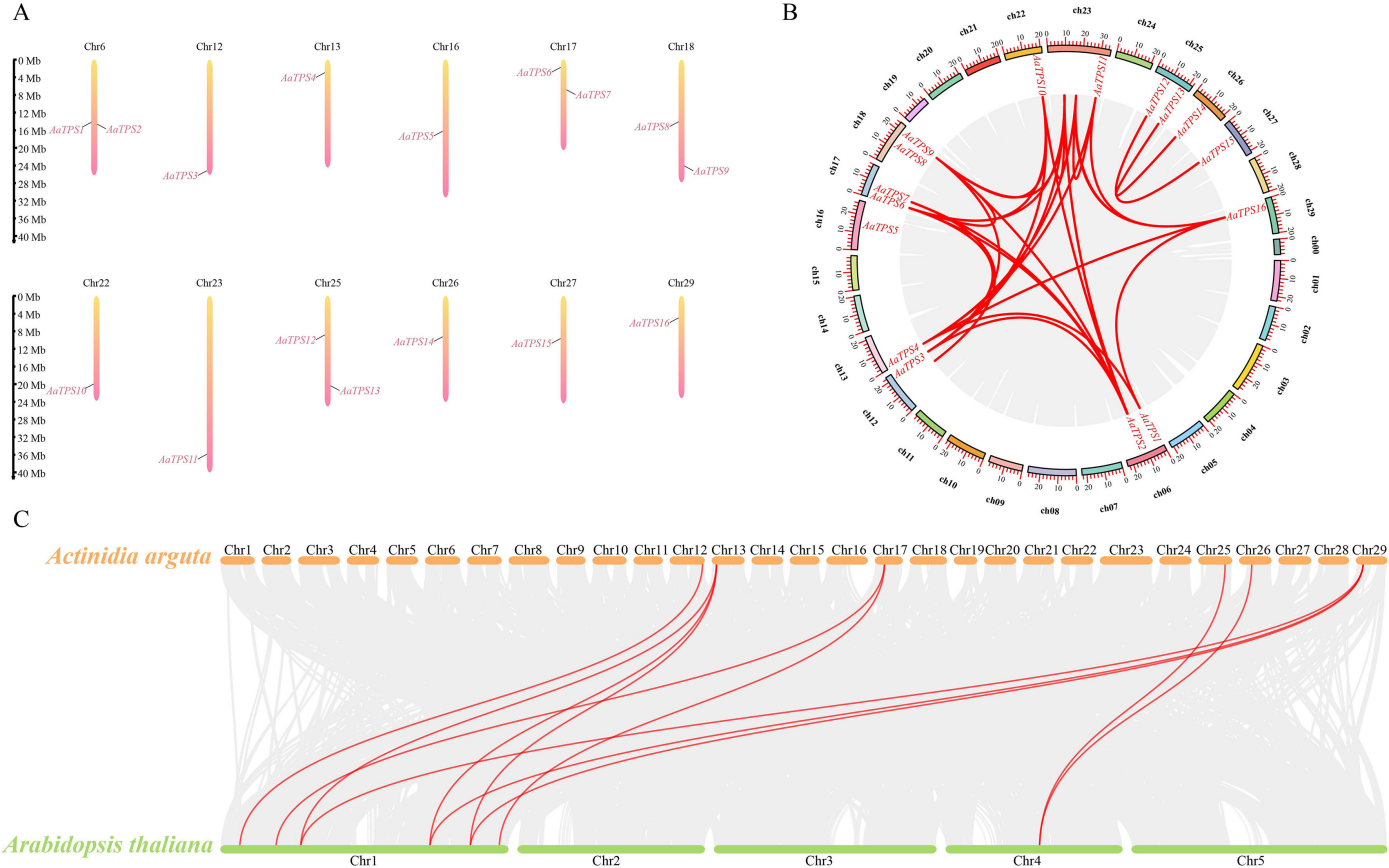
Chromosome localization **(A)**, syntenic analysis of *TPS* genes in *A. arguta* **(B)**, and syntenic analysis of *TPS* genes between *A. arguta* and *A. thaliana* **(C)**.

The 16 *AaTPS* genes were found to be unevenly distributed throughout 12 chromosomes, with different distribution patterns across Class I and Class II members, according to chromosomal localization analysis (**Figure 4A**). *AaTPS1* (Chr6) and *AaTPS7* (Chr17) did not form gene clusters and were found on separate chromosomes in the Class I group. In contrast, Class II members were found on 10 chromosomes, with two *AaTPS* genes present on Chr6, Chr17, Chr18, and Chr25. Remarkably, the distance of around 10.1 Mb between *AaTPS12* and *AaTPS13* on Chr25 suggests that they might have originated from a local duplication event. Overall, the *AaTPS* genes did not exhibit any recognizable gene clusters, indicating that the expansion of this gene family was more likely to be due to large-scale genome duplication events than tandem duplication. Additionally, the dispersed chromosomal placement raises the possibility that *AaTPS* genes have followed distinct evolutionary pathways and experienced functional diversification on many chromosomes.

Several *AaTPS* gene pairs with distinct collinear connections were found by intragenomic synteny analysis in *A. arguta* (**Figure 4B**). For example, gene pairs such as *AaTPS2*-*AaTPS3* and *AaTPS6*-*AaTPS9* showed strong synteny, suggesting that these genes may have arisen from duplication events during genome expansion. In addition, interspecies synteny analysis between *A. arguta* and *A. thaliana* revealed multiple collinear gene pairs with potential functional relevance (**Figure 4C**), including *AaTPS4*-*AtTPS8* and *AaTPS7*-*AtTPS2*. These conserved syntenic relationships further support the evolutionary conservation of the *TPS* gene family across species and provide a genetic basis for subsequent functional analyses of *TPS* genes in cold tolerance responses.

To investigate the evolutionary selective pressures acting on the *AaTPS* gene family, Ka/Ks analysis was performed for syntenic homologous gene pairs (**Table 4**). The results showed that all homologous gene pairs exhibited Ka/Ks ratios of less than 1, indicating that the *AaTPS* gene family has been predominantly subjected to purifying selection during evolution and that its core functions are highly conserved. Subsequent investigation showed that the synonymous substitution rate (Ks) varied greatly, from 0.128 to 2.844. Several duplication events that occurred at different evolutionary stages within the genome of *A. arguta* are reflected in this, which shows that the divergence periods of *AaTPS* homologous gene pairs range greatly. Conversely, the range of the nonsynonymous substitution rate (Ka) was very narrow (0.015-0.288), indicating that changes in amino acids across long-term evolution are tightly controlled. The gene pair *AaTPS1*-*AaTPS7* had a Ka/Ks ratio of 0.105, suggesting strong sequence conservation, in contrast to the *AaTPS3*-*AaTPS6* gene pair, which had a somewhat higher Ka/Ks ratio of 0.142. Even though this number is still much below 1, it suggests some adaptive evolution or functional divergence against the background of purifying selection. The Ka/Ks study reveals that the *AaTPS* gene family may have followed multiple evolutionary routes, allowing it to participate in *A. arguta* responses to abiotic stresses such as cold tolerance while preserving conserved core functions.

**Table 4:** Analysis of Ka/Ks selective evolution in the AaTPS gene family.

AaTPS_seq1	AaTPS_seq2	Ka	Ks	Ka/Ks
AaTPS1	AaTPS7	0.132713	1.260709	0.105269
AaTPS2	AaTPS10	0.073724	1.001663	0.073602
AaTPS2	AaTPS3	0.125005	2.843631	0.043960
AaTPS2	AaTPS6	0.129578	2.162824	0.059912
AaTPS2	AaTPS9	0.065241	0.798021	0.081754
AaTPS3	AaTPS10	0.133792	2.332137	0.057369
AaTPS3	AaTPS6	0.018219	0.128358	0.141937
AaTPS3	AaTPS9	0.130432	2.568629	0.050779
AaTPS4	AaTPS11	0.277886	1.923351	0.144480
AaTPS4	AaTPS16	0.037993	0.162426	0.233909
AaTPS6	AaTPS10	0.137699	1.949786	0.070623
AaTPS6	AaTPS9	0.133713	2.339132	0.057163
AaTPS9	AaTPS10	0.041605	0.182717	0.227703
AaTPS11	AaTPS16	0.287594	1.929371	0.149061
AaTPS12	AaTPS15	0.045528	0.236601	0.192424
AaTPS13	AaTPS14	0.014855	0.193712	0.076684

### 3.5 Cis-acting element analysis of promoters of the *AaTPS* gene family

**Figure 5:**
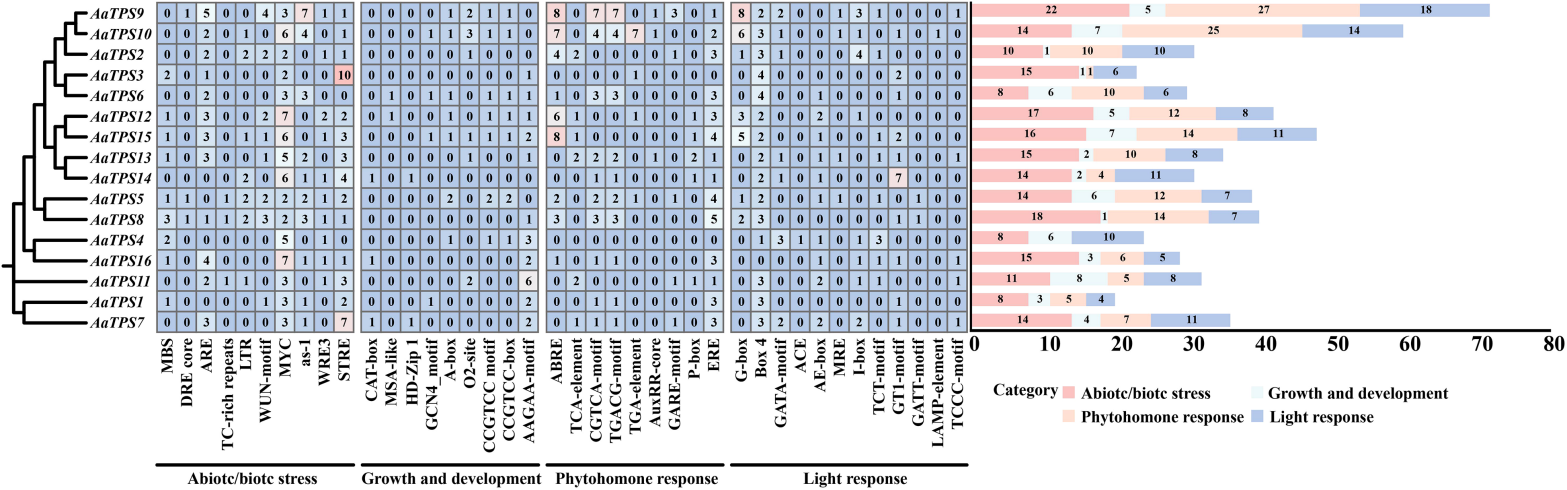
Predicted cis-elements of promotors in *AaTPS* gene family.

To further elucidate the potential functions and regulatory mechanisms of *AaTPS* genes, the 2,000 bp upstream promoter regions were analyzed, as these regions play crucial roles in gene regulation and functional expression. The results showed that the cis-acting regulatory elements identified in the promoters of the 16 *AaTPS* genes were mainly associated with light responsiveness, hormone regulation, abiotic stress responses, and circadian rhythm regulation (**Figure 5**). Notably, all 16 *AaTPS* genes contained light-responsive elements in their promoter regions. In addition, drought-inducible elements (MBS) and low-temperature-responsive elements (LTR) were identified in the promoters of 9 and 7 *AaTPS* genes, respectively, suggesting that members of the *AaTPS* gene family may participate in transcriptional regulation during drought and cold tolerance responses.

### 3.6 Expression patterns of the *AaTPS* gene family in cold tolerance

**Figure 6:**
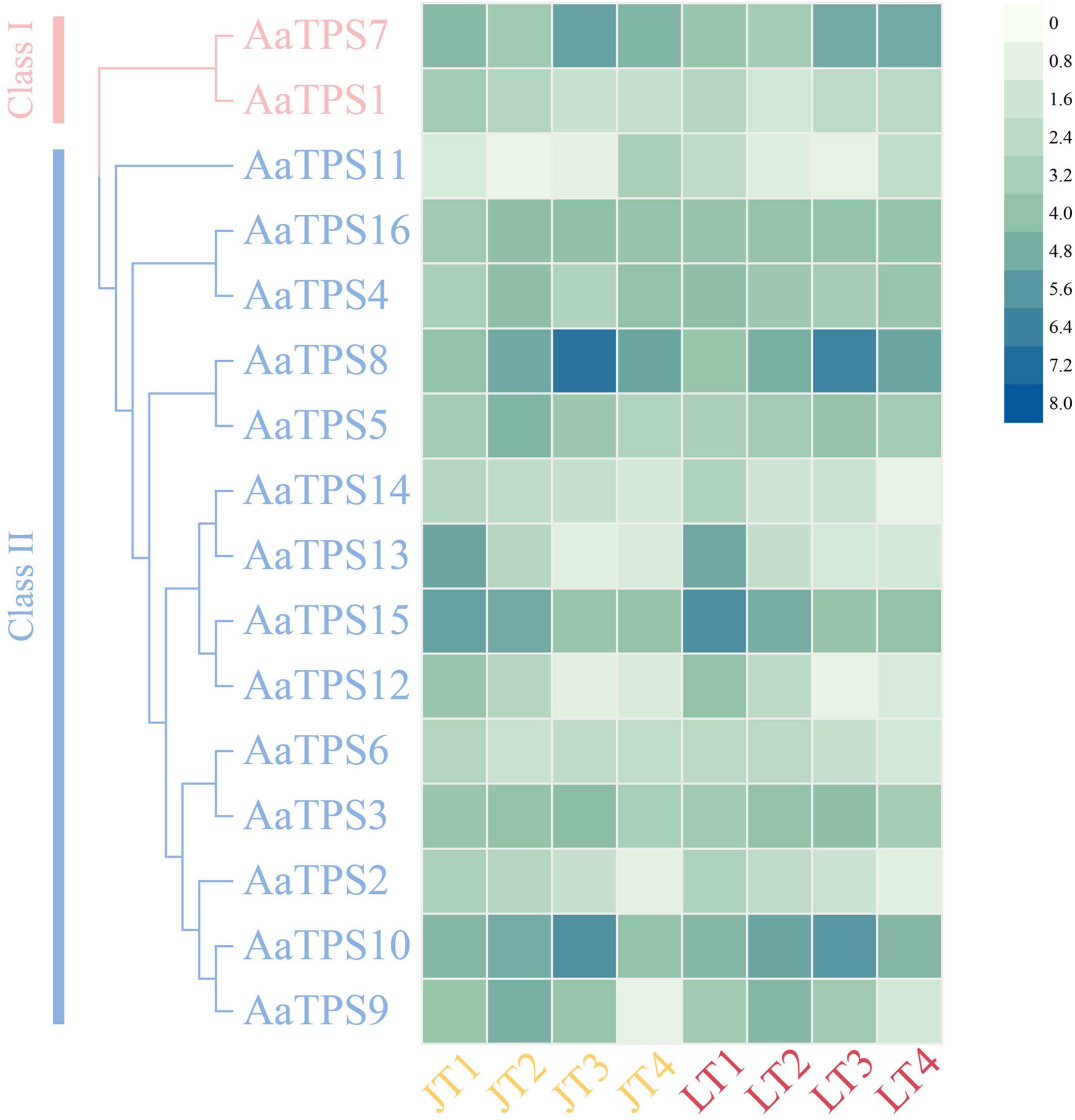
Expression pattern of AaTPSs in cold tolerance. The J and L respectively represent ‘Jialv’ and ‘Longcheng No.2’, T1-T4 represent different cold resistance stages, corresponding to 16 Sep, 6 Oct, 6 Nov and 6 Dec respectively. Light green and blue indicate low and high expression levels, respectively, based on normalized FPKM values.

To systematically investigate the expression characteristics of the *AaTPS* gene family in response to cold stress, transcriptome data obtained during the overwintering period from a cold-tolerant cultivar (‘Jialv’) and a relatively cold-sensitive cultivar (‘Longcheng No. 2’) were analyzed. The expression patterns of 16 *AaTPS* genes were examined, and a heatmap was generated to visualize their transcriptional dynamics (**Figure 6**). The results showed that most *AaTPS* genes displayed dynamic expression patterns across different sampling time points and between the two cultivars, suggesting that members of the *AaTPS* gene family may be involved in the response of *A. arguta* to low-temperature conditions.

Among them, *AaTPS8*, *AaTPS10*, and *AaTPS15* exhibited relatively high expression levels in both cultivars and showed increased expression in November and December during the overwintering period. In the cold-tolerant cultivar ‘Jialv’, *AaTPS8* and *AaTPS10* reached their highest expression levels in November and displayed higher expression than those observed in September and October, suggesting a cold-responsive expression pattern. In the cold-sensitive cultivar ‘Longcheng No. 2’, these genes also exhibited increased expression during the overwintering period, although their expression levels appeared to be lower than those in ‘Jialv’. In addition, *AaTPS1*, *AaTPS2*, *AaTPS6*, and *AaTPS14* exhibited relatively stable or low expression levels in both cultivars, with only minor fluctuations over time, suggesting that these genes may play constitutive or indirect roles in the response to cold stress.

**Figure 7:**
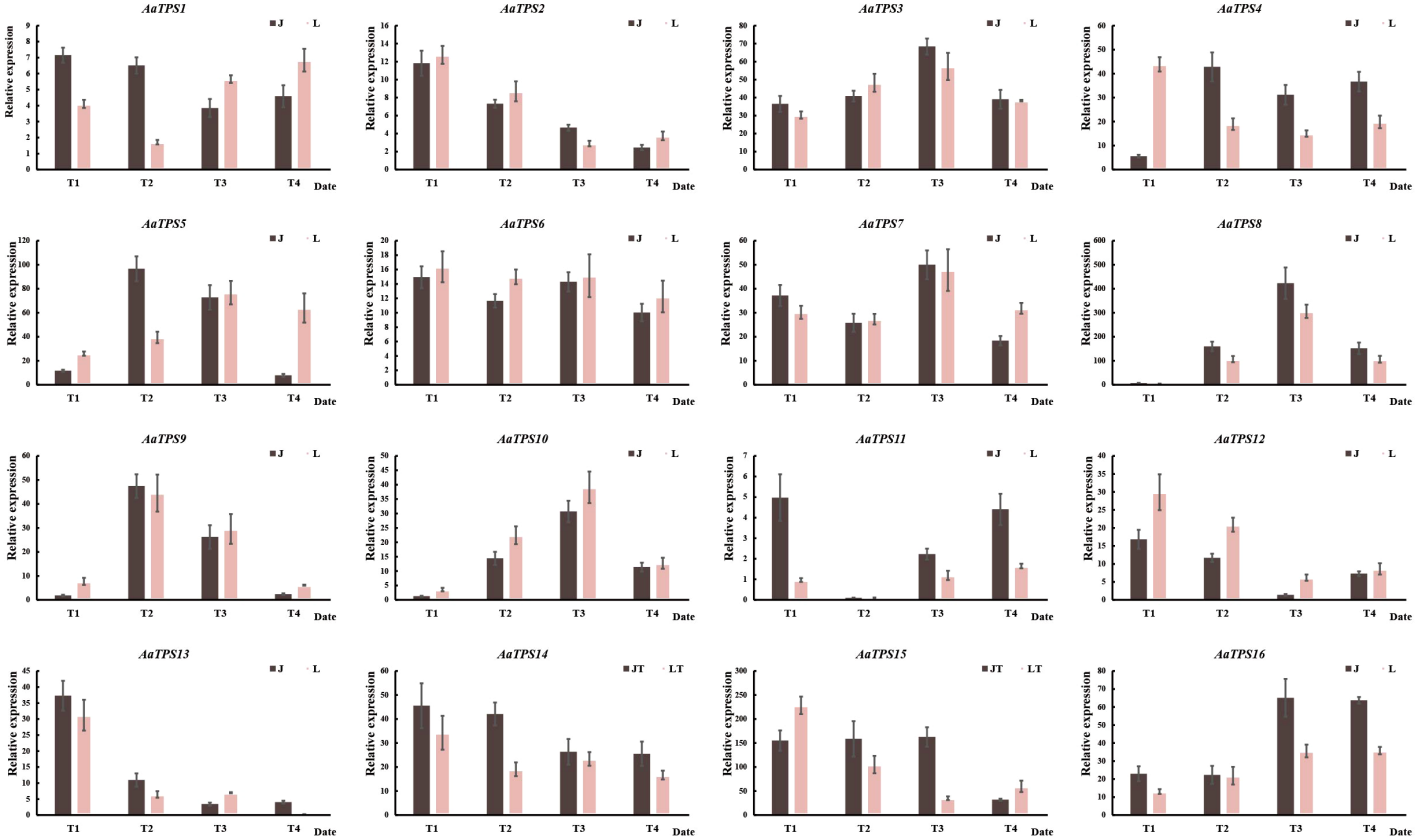
Relative expressions of *AaTPS* genes in cold tolerance.

To verify the transcriptome results, several *AaTPS* genes showing distinct expression patterns in the transcriptome analysis were selected for qRT-PCR validation. The expression patterns of these genes were analyzed in samples collected from different months in the two cultivars. The qRT-PCR results showed expression trends generally consistent with those obtained from the transcriptome analysis, supporting the reliability of the RNA-seq data (**Figure 7**).

### 3.7 Subcellular localization of analysis

**Figure 8:**
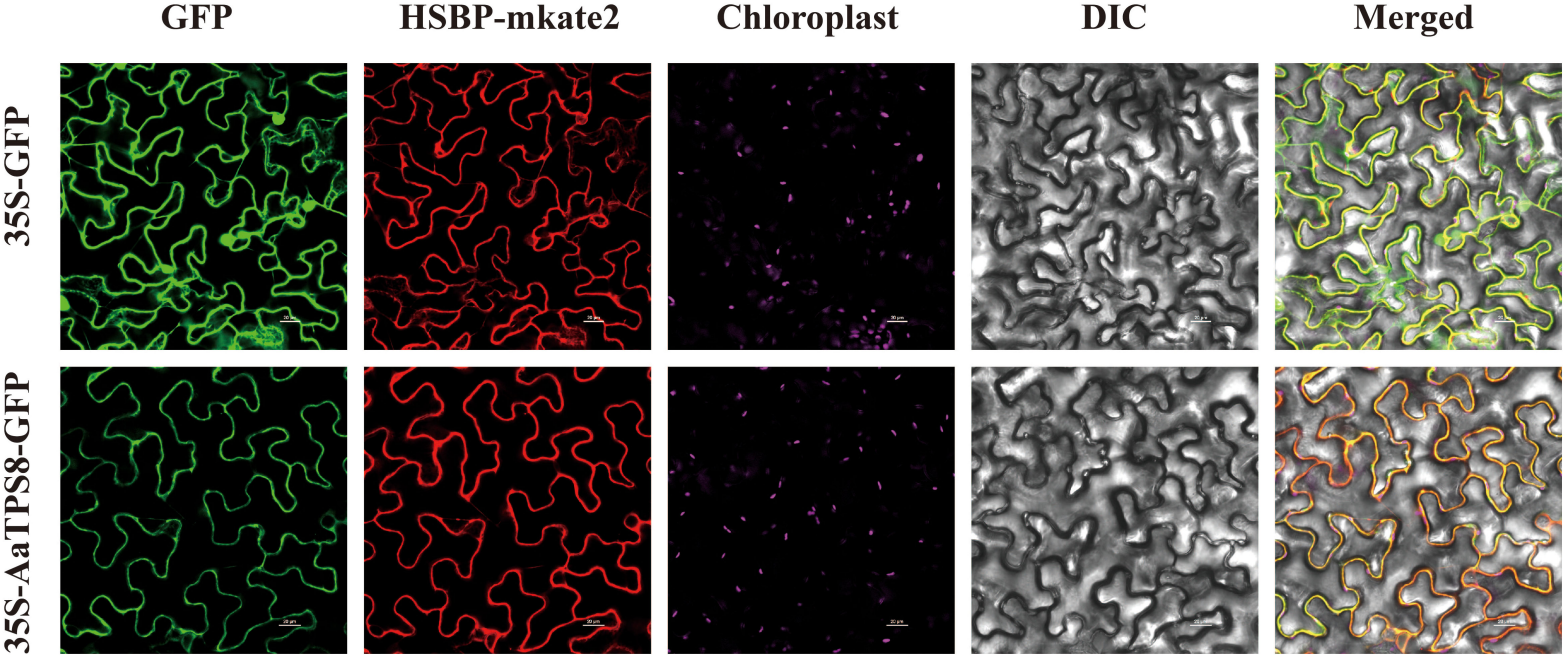
Subcellular localization of AaTPS8.

Based on multiple lines of evidence, including gene expression levels, comparisons of expression differences between cultivars, and the integrity of conserved functional domains, this study ultimately selected AaTPS8 for subsequent subcellular localisation analysis to identify its potential functional sites and mode of action. Although AaTPS10 and AaTPS15 showed increased expression during the later stages of cold acclimation, they exhibited relatively lower expression and indistinct cultivar differentiation, so no further validation was performed. Fluorescence observation of tobacco epidermal cells transiently expressing AaTPS8-GFP showed complete signal overlap between the fusion protein and cytoplasmic marker. No co-localization signals were found in other subcellular structures, which confirmed that AaTPS8 resides specifically in the cytoplasm (**Figure 8**).

## 4 Discussion

TPS is an essential rate-limiting enzyme in the metabolism of trehalose in plants. It is an essential step in the biosynthesis of trehalose, and its catalytic product T6P is an important signaling molecule that links the growth and development of plants, energy balance, and carbon metabolic status (Paul et al., 2020; Avidan et al., 2024; Fichtner, 2025; Rojas et al., 2023). According to accumulating evidence (Annunzaita et al., 2025; Göbel and Fichtner, 2023), T6P is placed at the nexus of sugar signaling, metabolic regulation, and developmental control and is critical for controlling the distribution of photosynthesis related assimilates, energy consumption, and growth choices. By combining carbon availability with growth regulatory networks, T6P level modification in crops can significantly affect how assimilated carbon is distributed among various metabolic pathways and organs, allowing plants to balance growth and metabolism in resource constrained environments (Liu et al., 2023; Zhai et al., 2021). More recent investigations have shown the role of the T6P-SnRK1 signaling pathway in regulating carbon allocation and stress adaption, notably in cold tolerance, drought, and salt stress conditions, where its regulatory effects are highly environmental dependent (Nunes et al., 2013; Paul et al., 2018; Baena-González and Lunn, 2020). However, comprehensive discovery of the *TPS* gene family and clarification of its regulatory roles in cold tolerance remain largely unexplored in permanent woody fruit trees like *A. arguta*, in contrast to model plants and annual crops.

The genome of *A. arguta* contained 16 *AaTPS* genes in total, which is comparable to the *TPS* gene family size reported in several representative plant species, including apple (Du et al., 2017), peach (Fan et al., 2024), Populus (Gao et al., 2021), cucumber (Dan et al., 2021), soybean (Liu et al., 2026), and watermelon (Yuan et al., 2021), indicating that the *TPS* gene family size is relatively conserved across these species. The coexistence of structural conservation and diversification within this gene family is demonstrated by the fact that, despite differences in amino acid length, molecular weight, and isoelectric point, the overall physicochemical characteristics of AaTPS proteins were mainly in agreement with TPS proteins identified in Arabidopsis, rice, and apple. In accordance with the functional properties of TPS as intracellular soluble enzymes involved in sugar metabolism and signaling regulation, all AaTPS proteins were predicted to be hydrophilic, and the majority were categorized as unstable proteins (Lunn et al., 2014; Du et al., 2017).

The *TPS* gene family has been shown to be evolutionary conserved throughout angiosperms, as evidenced by the obvious classification of *AaTPS* genes into Class I and Class II subfamilies by phylogenetic analysis. This pattern is very similar to those in maize (Acosta-Pérez et al., 2020), rice (Zang et al., 2011), and Arabidopsis (Leyman et al., 2001). Previous studies have shown that Class I *TPS* members play important roles in primary metabolism as well as plant growth and development. They also maintain their full catalytic domains and display unambiguous TPS enzymatic activity. In contrast, Class II *TPS* members primarily engage in signal transduction and stress responsive regulation, although they frequently show little to no enzymatic activity (Lunn et al., 2014; Avonce et al., 2006; Yang et al., 2012). Among the 16 *AaTPS* genes identified in *A. arguta*, only two belong to Class I, whereas the remaining fourteen belong to Class II, indicating a clear predominance of Class II members. This subfamily composition is consistent with that reported in several representative plant species, including *A. thaliana*, poplar, and other horticultural crops (Leyman et al., 2001; Vandesteene et al., 2010; Gao et al., 2021; Du et al., 2017), suggesting that the overall organization of the *TPS* gene family has remained evolutionarily conserved. The predominance of Class II *TPS* genes suggests that *A. arguta* may have evolved an enhanced regulatory capacity to cope with complex environmental conditions.

Chromosomal localization studies revealed that *AaTPS* genes were unevenly dispersed over 12 chromosomes, with significant physical gaps between genes on the same chromosome and no clear tandem duplication clusters. This distribution pattern is consistent with results from cucumber (Dan et al., 2021), watermelon (Yuan et al., 2021), and apple (Du et al., 2017), indicating that whole-genome or segmental duplication events, rather than tandem duplication, were the primary drivers of the *AaTPS* gene family’s expansion. This finding was supported by synteny analysis, which revealed that some *AaTPS* genes shared strong collinear linkages across several chromosomes, indicating that they came from previous genome duplication events. According to Ka/Ks analysis, all homologous *AaTPS* gene pairs had Ka/Ks ratios less than 1, indicating that purifying selection had a significant impact on the gene family. Evolutionary studies of *TPS* gene families in other plant species back with this conclusion. The relatively low Ka values indicate strong functional constraints on AaTPS protein sequences, however the wide range of Ks values indicates that these genes resulted from duplication events that occurred at distinct evolutionary time scales. This evolutionary trend provides a molecular underpinning for functional diversity at the transcriptional regulatory level by combining variable duplication timing with high sequence conservation.

Conserved domain analysis indicated that all AaTPS proteins had the *TPS* family’s signature PF00982 and PF02358 domains, indicating high structural conservation. This finding is consistent with previous research on peaches, apples, rice, and Arabidopsis (Leyman et al., 2001; Acosta-Pérez et al., 2020; Zang et al., 2011; Fan et al., 2024). Nonetheless, there were differences in protein length and domain integration across AaTPS members, with some proteins having fragmented domain topologies, indicating potential functional attenuation or transition throughout evolution. Gene structure and conserved motif analyses revealed increased structural heterogeneity within the *AaTPS* family. Genes with closer evolutionary ties have comparable exon-intron structures and motif compositions, a phenomenon commonly observed in *TPS* and other gene families across plant species (Du et al., 2017; Fan et al., 2024). In contrast, certain *AaTPS* genes have simpler gene architectures that were postulated to promote quick transcriptional responses or regulatory activities under stress circumstances rather than acting as key metabolic enzymes (EH et al., 2025; Guo et al., 2025; Morabito et al., 2021).

Cis-acting element analysis revealed that the promoter regions of *AaTPS* genes were enriched with light-responsive elements, indicating that light signals may play a major role in basal regulation. Furthermore, several *AaTPS* promoters had cis-elements related with drought and cold stress, such as MBS and LTR elements, indicating that they might respond to abiotic stress signals. Previous research has shown that changes in cis-element composition are an important biological foundation for functional distinction between members of the same gene family during stress reactions (Cui et al., 2023; Schmitz et al., 2021).

Transcriptome analysis revealed distinct expression patterns of *AaTPS*s across different sampling periods and between the two cultivars during the overwintering period. Notably, during the time of steadily rising cold tolerance, the cold-tolerant cultivar generally exhibited higher expression levels of several *AaTPS*s than the cold-sensitive cultivar. This expression pattern is consistent with previous transcriptomic studies of cold adaptation in *A. arguta* and other woody plants. *AaTPS8*, *AaTPS10*, and *AaTPS15* all showed consistently higher expression levels during seasonal cold acclimation, suggesting that these genes may participate in the regulatory network underlying cold acclimation. Among them, *AaTPS8* may represent a promising candidate gene associated with seasonal cold acclimation. The qRT-PCR results showed expression trends consistent with the transcriptome data, supporting the reliability of the RNA-seq analysis.

By combining evolutionary traits, structural properties, cis-regulatory element distribution, and expression patterns, the present study suggests that the *AaTPS* gene family may aid *A. arguta* adaptation to cold tolerance by modulating T6P signaling and its related metabolic and regulatory networks. Previous studies have shown that T6P not only maintains energy balance by inhibiting the SnRK1 signaling pathway, but also activates the target of rapamycin (TOR) signaling pathway under carbon-sufficient conditions, enhancing plant growth and metabolic activity (Fichtner and Lunn, 2021; Morales-Herrera et al., 2023, 2024). Based on previous studies and the expression profiles observed in the present study, Class I *TPS* genes may primarily contribute to maintaining carbon metabolism and energy homeostasis under low-temperature conditions, whereas Class II *TPS* genes may play a greater role in signal transduction and transcriptional regulation, potentially improving *A. arguta* cold tolerance. These findings suggest that different *AaTPS* subfamilies may function cooperatively in the response to low-temperature conditions. Although the specific biological functions of individual *AaTPS* genes require further experimental validation, the present study provides a useful foundation for further investigation of the molecular mechanisms underlying cold tolerance in perennial woody plants such as *A. arguta*.

The subcellular localization results indicate that the AaTPS8 protein is primarily localized in the cytoplasm, suggesting that it may primarily participate in processes related to trehalose metabolism within the cytoplasm (Vandesteene et al., 2010). Previous studies have shown that TPS proteins play a crucial role in plant sugar metabolism, signal transduction, and stress response, and the cytoplasm is an important site for sugar synthesis and energy metabolism. Therefore, this localization result is consistent with the biological function of the TPS protein. Furthermore, T6P produced through TPS mediation not only participates in trehalose synthesis but also serves as an important signaling molecule regulating plant growth, development, and stress resistance (Ponnu et al., 2011). The results of this study provide a theoretical basis for further functional characterization of AaTPS8 in *A. arguta*. Taken together, *AaTPS8* should be regarded as a candidate gene associated with seasonal cold acclimation, and its role in cold tolerance requires further validation through physiological, biochemical, and genetic approaches.

## 5 Conclusion

In this study, 16 *TPS* gene family members were identified in the *A. arguta* genome and classified into Class I and Class II based on phylogenetic analysis, with members within the same subfamily exhibiting similar structural features. Synteny and evolutionary analyses revealed that *AaTPS* genes underwent duplication events in the *A. arguta* genome and have been subjected predominantly to purifying selection during evolution. Expression analysis demonstrated that *AaTPS8* exhibited strong transcriptional responses during seasonal cold acclimation, making it a candidate gene associated with seasonal cold acclimation. In addition, the subcellular localization results indicate that the AaTPS8 protein is primarily localized in the cytoplasm, suggesting its potential involvement in trehalose metabolism and stress response regulation processes within the cytoplasm. Collectively, these findings provide a theoretical foundation for elucidating the evolutionary characteristics and potential functions of the *TPS* gene family in *A. arguta* in cold tolerance and lay the groundwork for future molecular breeding and functional gene mining aimed at improving cold tolerance in *A. arguta*.

## Data Availability

The original contributions presented in the study are included in the article/supplementary material. Further inquiries can be directed to the corresponding authors.
